# Findings From a Community Survey of Individuals Who Engage in Pup Play

**DOI:** 10.1007/s10508-021-02225-z

**Published:** 2022-04-25

**Authors:** Liam Wignall, Mark McCormack, Taylor Cook, Rusi Jaspal

**Affiliations:** 1 Department of Psychology, Poole House, Bournemouth University, Poole, BH12 5BB UK; 2 School of Humanities and Social Sciences, University of Roehampton, London, UK; 3 Nerdy Doggy, Melbourne, Australia; 4 Department of Psychology, University of Brighton, Brighton, UK

**Keywords:** BDSM, Kink, Pup Play, Sexuality, Social identity

## Abstract

This study presents findings from a community survey on pup play. Pup play is a kink activity and a form of role play that is growing in popularity internationally, and gaining increasing attention in sexology, yet prior research on pup play has almost entirely employed qualitative methods and primarily involved gay and bisexual men. Using survey data of 733 pup play participants primarily from the US, but also internationally, this study reports on the demographics of participants, how they engage in pup play, its social and sexual elements, and how it relates to social identity and mental health. Unique pup names and identifying with breeds of dogs were used to foster a sense of individuality within pup play, while the majority of participants owned and wore gear when engaging in pup play. We also found significant associations between being younger and identifying as a pup. Most participants reported that pup play improved their mental health. Binary logistic regression analyses indicated that having a mental health diagnosis was associated with identifying with a more social style of pup play and self-reporting the mental health benefits of pup play. We find that the conceptualization of pup play in the existing literature to be accurate to this international sample and highlight areas where further research is needed, alongside limitations of the study.

## Introduction

Pup play, or puppy play, refers to a form of role play in which adult humans mimic the behavior of mostly young dogs. Individuals tend to imitate the posture of a dog and wear a collar and other gear associated with owning a dog (Wignall & McCormack, 2017). The activity can occur alone, with other “pups” or with a handler. Practitioners tend to adopt a submissive role, but there can be power hierarchies among pups. Most practitioners describe it as a sexual/erotic activity, yet it also has important social functions, allowing individuals to interact in tactile and playful ways, and providing a common interest leading to the development of communities, subcultures and distinctive social identities. Pup play practitioners value the social and sexual intimacy of pup play, the ability to relax and explore different headspaces that it provides (Wignall & McCormack, 2017), and pup play is an important component of many practitioners’ social identities and social networks (Langdridge & Lawson, 2019). Communities have formed around pup play, and it exists as a distinct subculture within the broader BDSM (Bondage/Discipline, Domination/Submission and Sadism/Masochism) subculture (Wignall, in press).

Cultural interest in pup play has grown over the past decade, with significant media attention in the form of mainstream news articles (e.g., Montgomery, 2019) and television documentaries, as well as books published by those involved in pup play (Daniels, 2006; St. Clair, 2015). In the academic literature, pup play has been recognized in broader research on BDSM cultures as distinct from other kink behaviors, identities and communities (e.g., Franklin et al., 2020; Paasonen, 2018). For example, in their survey of men at a kink-focused and LGBTQ Pride event, Moskowitz et al. (2011) found of people who possessed a Leather-focused identity, 51 people (7.8%) identified as pups. Yet, little research has had a primary focus on understanding pup play.

Pup play was first considered as the primary research focus in 2017 (Wignall, 2017; Wignall & McCormack, 2017), spurring subsequent academic interest (Boyd, 2018; Langdridge & Lawson, 2019; Lawson & Langdridge, 2020; Wignall, in press). This emergent body of research has sought to: classify pup play as a sexual, social and community activity, using concepts such as subculture, neotribe and leisure sex; trace its history; and understand its social dynamics, situating pup play as a form of BDSM and distinguishing it from zoophilia (cf. Aggrawal, 2011). However, the research on pup play is currently restricted to qualitative studies, predominantly with gay and bisexual young men. In this study, we present quantitative analysis from a community survey of pup play practitioners mostly from the US, but also internationally, to examine practices of pup play and to test the applicability of the qualitative research more generally, enhancing our understanding of this increasingly popular kink activity.

### Kink Subcultures and Social Change

Pup play has developed and consolidated as a sexual subculture and kink activity in the context of broader cultural shifts within kink subcultures and communities. Kink is a collection of diverse erotic or sexual practices, relationships and identities, normally oriented around power exchange, pain and role play (see Sprott & Williams, 2019; Wignall, in press). Early examples of kink subcultures often mirrored the dynamics of sexual subcultures more generally (Weinberg, 1978), with individuals coming together based on common sexual interests or identities (Plummer, 1995; Weeks, 1977). These communities and subcultures provided the opportunity to experience positive distinctiveness in a broader context of cultural marginalization (see also Graham et al., 2016; Newmahr, 2011). This closeness within subcultures, coupled with societal stigma and legal prejudice toward kink (Rubin, 1991), often led to kink subcultures being secretive and hard to access, both for people wanting to participate in kink and for academic researchers (Weinberg, 2006).

Emphasizing this, kink subcultures often have sophisticated rules and social norms, such as how to interact with others in kink environments (e.g., Bauer, 2014; Stiles & Clark, 2011); limitations on how one can gain access and membership to a subculture (Rubin, 1981; Weinberg, 2006); particular notions of how kink should be practiced (Downing, 2007; Williams et al., 2014); and debates around the boundaries what is considered kink (Damm et al., 2018; Simula, 2019a). Such protocols and restrictions relating to kink subcultures and ways of practicing kink were often labeled “Old Guard” with more recent examples of kink subcultures forming the “New Guard.” However, as Rubin (1998) highlights, this shift is complex and has occurred over a long period of time, with examples of Old and New Guard ways of engaging in kink visible in contemporary kink subcultures.

Over the last few decades, notable shifts in kink subcultures have occurred. Kink has become more visible in the public sphere (Weiss, 2006), moving from underground subcultures to public and semipublic spaces. For example, kink is present in varied forms at pride parades (Ammaturo, 2016); the movie *Fifty Shades of Grey* attracted global media attention (Drdová & Saxonberg, 2020); reporting on kink has appeared in leading broadsheet newspapers (Montgomery, 2019) and throughout pop culture more generally (Khan, 2017). The internet has also provided individuals with easier ways to engage in kink subcultures and find others to explore kink interests with (e.g., Randall & McKee, 2017; Simula, 2019a; Wignall, in press).

The increased ease with which individuals can explore their kink interests and access kink communities, particularly through the internet (Cascalheira et al., 2021b; Denney & Tewksbury, 2013; Döring, 2009), has allowed a new generation of people to explore kink interests (Zambelli, 2017). Indeed, people can explore kink at a more comfortable pace, eschewing the formality and protocol associated with Old Guard ways of practicing kink (Wignall, in press). Given the focus on the playful nature of kink within pup play (Langdridge & Lawson, 2019), alongside the ability to explore different kinks simultaneously (Wignall & McCormack, 2017), pup play provides a unique way of exploring different kink activities.

These changes have formed part of a broader liberalization of attitudes toward various forms of consensual sexual activity (Frank & McEneneany, 1999; Loftus, 2001), with sustained growth in acceptance of non-marital sex, oral and anal sex and same-sex romantic relationships (Habel et al., 2018; Twenge et al., 2015, 2016). These changes have notable limits, with sizable minorities still objecting to such practices, the gendered double standard related to casual sex, and evidence that kink is still stigmatized (Thompson et al., 2018), causing some to argue that practices of “leisure sex” have undergone a process of normalization rather than liberalization (McCormack et al., 2021).

### Research on Pup Play

In the first academic article on pup play, Wignall and McCormack (2017) conducted 30 interviews with White gay and bisexual men in the UK who engaged in pup play. They provided rich description of pup play and routes into the activity, while demonstrating it as a kink activity (Weinberg et al., 1984). They also argued it should be understood as a leisure activity, mirroring how the leisure framework has been applied to other kink activities (e.g., Newmahr, 2010; Prior & Williams, 2015; Williams et al., 2016). Wignall and McCormack also argued pup play could be both a sexual and social activity, depending on the setting and context in which it is practiced (see also Simula, 2019b). They emphasized the relaxed rules in how one can engage pup play, highlighting the personal and playful nature of pup play, and the sharp contrast to the more traditional ways of engaging in kink subcultures (see Rubin, 1991).

Drawing on the same data set, Wignall (2017) documented how pup subcultures consolidated through using Twitter to create an online community. Wignall demonstrated how pup play mirrors more recent trends within kink subcultures through the development of online communities (e.g., Colosi & Lister, 2019). These online communities are markedly different from older kink subcultures which were primarily organized around subcultural community venues (Steinmetz & Maginn, 2014). While sociosexual networking sites already exist for kinky individuals (e.g., Zambelli, 2017), Wignall (2017) argued Twitter was preferred by participants due to its familiarity, the ability to create multiple profiles, and the existence of a mobile app. The distinct ways pups engaged with the platform, including helping to interact with other pups around the world, led to the creation of what Wignall called “Pup Twitter”. The social identity aspects of pup play were consolidated by using Twitter handles that tended to be the word “Pup” followed by the person’s pup name.

Building on Wignall and McCormack’s work, Langdridge and Lawson (2019) conducted a survey into the experiences of individuals who engage in pup play to explore underlying psychological motivations behind the activity. Using a survey of 68 pup play practitioners and follow-up in-depth interviews with 25 participants, their sample contained more geographical diversity. In the survey, there were 44 “European”, 21 “North American” and three unattributed participants; in the interviews, 21 “European”, two “North American” and two Australian individuals participated. However, while the breakdown of European nationalities is not provided for the survey, 18 of the 21 “European” interviewees are from the UK—suggesting that a UK bias exists in this study as well.

Langdridge and Lawson (2019) adopted a phenomenological approach and identified five main rationales for engagement in pup play: sexual pleasure; relaxation/therapy and escaping from self; adult play and vibrant physicality; extending and expressing selfhood; and relationship and community benefits. They also identified the importance of community within pup play, describing it as an inherently relational activity in which identities are often co-produced with other pups and handlers. Broadly supporting the initial conceptualization of pup play (Wignall & McCormack, 2017), Langdridge and Lawson also drew attention to pup play as a form of relaxation and therapy that can enable escape from the stresses of daily life. This is similar to the finding that kink may have therapeutic qualities (Easton, 2007; Lindemann, 2011), particularly as a way of dealing with past trauma (Thomas, 2020). Individuals may reframe their traumatic experiences, strengthen their sense of self and reclaim power through engagement in kink (Cascalheira et al., 2021a, 2021b). Several participants in Langdridge and Lawson’s (2019) study reported feeling mental health benefits from participation in pup play, but there is a lack of broader evidence regarding perceptions of mental health and participation in pup play.

Drawing on the same data set, Lawson and Langdridge (2020) also used participants’ narratives alongside pup community texts (e.g., St Clair, 2015) and research into kink subcultures (e.g., Weiss, 2011) to outline the cultural history of pup play. They traced the antecedents of pup play in gay leather communities of the 1970s and 1980s, describing how contemporary pup play (with a focus on play and ability to explore the self) developed from more traditional Old Guard styles of dog/slave play. Drawing on postmodern-subcultural theory, they applied Irwin’s (1973) model of scene evolution to demonstrate how there has been an expansion in the popularity of pup play. With this expansion comes a corruption of the scene, they argued, where new members engaging with a scene and its norms but start to manipulate these norms to the distaste of more established members.

An important contribution was their discussion of the role of the handler in pup play (Lawson & Langdridge, 2020), describing how a handler held a similar role to dominants/masters in kink settings. A handler often has the responsibility of looking after a pup, carrying the pups’ paraphernalia or gear (e.g., toys, clothing, harness), providing training on how to behave more like a dog, and generally interact with their pups. Wignall (in press) has also documented that as pups age, some begin to transition into the handler role to mentor new pups, yet little is known about the handler role beyond this.

Research into pup play has been qualitative and conducted predominantly with gay and bisexual men in the UK. As Wignall and McCormack (2017) called for in their original article, research that is quantitative in nature and with participants from primarily outside the UK is needed to provide a better understanding of the dynamics of pup play internationally and test the generalizability of the qualitative findings to a broader population. While research has begun to incorporate pup play into understandings of BDSM and queer intimacies more generally (Fedoroff, 2019; Hammack et al., 2019; Jaspal, 2019; Simula, 2019a; Tiidenberg & Paasonen, 2019), these contributions have been predominantly conceptual rather than empirical, connecting pup play with other activities, identities and communities, rather than providing new research on pup play.

The purpose of this study is to address these limitations by using a large community survey of pup play to provide quantitative analysis of pup play from a primarily US-based sample and to explore associations between certain characteristics of pup play, including age, role and mental health.

## Method

Data come from a 2019 survey designed and organized by pupplay.info and *Nerdy Doggo—*an Australian nonprofit organization which provides information about pup play and its history. The title of the survey was “2019 Nerdy Doggo Community Survey”, with the survey “helping the [pup] community know where it is in 2019 and to help PAH (pups and handlers) know what the community is focused on to better provide support and resources to its members.” Anybody involved in pup play was invited to participate, including handlers. The survey was distributed across various social media platforms (e.g., Twitter; Facebook; FetLife; Telegram; WhatsApp), focusing on groups/accounts dedicated to pup play. The survey was also disseminated through snowball sampling. The survey was hosted on Google forms, and focused on the experiences of those involved in pup play (pups and handlers). Bournemouth University, UK, provided ethical approval for secondary analysis of the survey findings.

### Participants

Inclusion criteria specified that participants needed to be at least 18 years old and involved in pup play in some capacity, as a pup, handler or both. Overall, 747 individuals completed the survey. However, 14 participants were under the age of 18 and so were excluded from the study in line with ethical approval. The final total was 733 participants.

### Measures

The focus of the survey was to understand how pup play occurs within different communities so that *Nerdy Doggo* could provide suggestions to PAH community groups about what support people involved in pup play required. As such, not all the data are included in this paper (e.g., eating habits). The survey was divided into the following sections:

#### Demographics

Participants were asked to indicate via tick box if they were a: pup; handler; pup and handler (pup focused); or a handler and pup (handler focused). Geographical region and age were asked via tick boxes, with the responses in Table 1. Free text boxes were used to provide gender and sexuality. Participants were asked for how many years they have identified as pup, handler or both, and how many years they have participated in the pup community (free text box).

**Table 1:** Age and geographical breakdown of participants

Age	Handler	Pup	Pup & Handler	Total
18–20	1 (0.14%)	52 (7.09%)	14 (1.91%)	67 (9.14%)
21–30	14 (1.91%)	280 (38.20%)	65 (8.87%)	359 (48.98%)
31–40	25 (3.41%)	122 (16.64%)	36 (4.91%)	183 (24.97%)
41–50	24 (3.27%)	46 (6.28%)	16 (2.18%)	86 (11.73%)
51–60	5 (0.68%)	23 (3.14%)	4 (0.55%)	32 (4.37%)
61 +	2 (0.27%)	4 (0.55%)	0 (0%)	6 (0.82%)
Total	72 (9.82%)	527 (71.90%)	135 (18.42%)	733 (100%)
*Geographical Region*				
Africa	2 (0.27%)	2 (0.27%)	0 (0%)	4 (0.55%)
Asia/India	1 (0.14%)	3 (0.41%)	0 (0%)	4 (0.55%)
Europe	3 (0.41%)	79 (10.78%)	15 (0.20%)	97 (13.23%)
North America	51 (6.96%)	356 (48.57%)	104 (14.19%)	511 (69.71%)
Oceania	14 (1.91%)	85 (11.60%)	14 (1.91%)	113 (15.42%)
South America	0 (0%)	2 (0.27%)	2 (0.27%)	4 (0.55%)
Total	71 (9.69%)	527 (71.90%)	135 (18.42%)	733 (100%)

Percentage totals may total 100% due to rounding

#### Personal Identity

Likert scales were used to attest to the importance of certain features of pup play—the scales were 1–5, with 1 equating to “100% not important” and 5 equating to “100% important”. Questions asked about the importance of gear/equipment in expressing a pup play identity; personal identification with the pup/handler role; being social with pup play; public exposure with pup play; private activities with pup play; sexual activities with pup play; traditions with pup play; spirituality with pup play; sexual identity with pup play. Participants were asked if they owned gear related to their pup play (yes/no); if they had a name related to their pup play (yes/no); if they were a pup, if they identified with a breed of dog (yes/no); how many hours per week they participated in pup play (free text); if they had a tattoo relating to their pup play (yes/no); and if they identified with the term “pup” or “handler” as portrayed in the community (yes/no).

#### Health and Well-Being

Participants were asked if they had any mental health diagnosis (yes/no/rather not say), if pup play improved their mental health (yes/no/rather not say), and if they had sustained any injuries during pup play (no/ yes–minor/ yes–major). Participants were also asked if they were diabetic, gluten intolerant, lactose intolerant, vegan or vegetarian (tick all that apply).

#### Chosen Family and Community

Participants were asked if they attended pup play events at public venues (yes/no), if they had attended a pup play mosh (yes/no) or attended a pup play workshop (yes/no), if they belonged to a pack/chosen family (yes/no), whether they were all living in the same country if they did belong to a pack (yes/no), and how many members (free text box). Participants were also asked to rate on Likert scales the importance of attending a pup mosh, attending a workshop, and (if they belonged to a chosen family/pack) the importance of their chosen family/pack. Scales were 1–5, with 1 equating to “100% not important” and 5 equating to “100% important”. Participants were also asked if they did not belong in a chosen family/pack, would they want to join one in the future (yes/no/maybe).

#### Gear and Other Fetishes

Participants were presented with a list of gear and asked to indicate which they owned, and which they used during pup play (tick box). The list included: headgear, harnesses, protective gear, handgear, collars, leashes, tails, toys, insertable toys and clothing. Participants were asked if the color of the gear was important to them (yes/no/I don’t own gear). Finally, a free text box was provided for participants to state what fetishes they actively participate in during pup play sessions.

### Data Analysis

All data analysis was conducted using SPSS (v.23). Descriptive analyses were conducted to document the frequency of behaviors. Content analysis was used to analyze qualitative data in free text boxes to provide frequency data based on common themes. A chi-square test of independence was conducted to explore associations between age and specified role. Binary logistic regression analyses were also conducted to explore links between aspects of mental health and engagement in pup play.

## Results

### Demographics

Most of the sample were aged 18–30 years (*n* = 426, 58.12%), with 307 participants (41.88%) older than 30. There was a geographical spread with participants from North America (*n* = 511, 69.71%), Oceania (*n* = 113, 15.42%), Europe (*n* = 97, 13.23%), South America (*n* = 4, 0.54%), Africa (*n* = 4, 0.54%) and Asia (*n* = 4, 0.54%). Table 1 provides a full breakdown of age and geographical regions. The majority of the sample consisted of men (*n* = 577, 78.72%), but also included women (*n* = 57, 7.78%) and nonbinary participants (*n* = 43, 5.86%). Most participants were gay/lesbian (*n* = 472, 64.39%), with smaller numbers of bisexual (*n* = 104, 14.19%), pansexual (*n* = 63, 8.59%) and straight (*n* = 22, 3.00%) individuals. Table 2 describes the full gender/sexuality distribution of participants.

**Table 2:** Gender and sexuality distribution of participants

	Female	Male	Nonbinary	Transgender	Other	Total
Asexual	2 (0.27%)	5 (0.68%)	3 (0.41%)	0 (0%)	2 (0.27%)	12 (1.64%)
Bisexual	18 (2.46%)	69 (9.41%)	6 (0.82%)	5 (0.68%)	6 (0.82%)	104 (14.19%)
Demisexual	0 (0%)	5 (0.68%)	2 (0.27%)	1 (0.14%)	0 (0%)	8 (1.09%)
Gay	2 (0.27%)	442 (60.30%)	9 (1.23%)	5 (0.68%)	7 (0.95%)	465 (63.44%)
Lesbian	5 (0.68%)	0 (0%)	2 (0.27%)	0 (0%)	0 (0%)	7 (0.95%)
Other	0 (0%)	7 (0.95%)	3 (0.41%)	2 (0.27%)	3 (0.41%)	15 (2.05%)
Pansexual	13 (1.77%)	25 (3.41%)	8 (1.09%)	12 (1.64%)	5 (0.68%)	63 (8.59%)
Queer	5 (0.68%)	16 (2.18%)	10 (1.36%)	1 (0.14%)	5 (0.68%)	37 (5.05%)
Straight	12 (1.64%)	8 (0.09%)	0 (0%)	1 (0.14%)	1 (0.14%)	22 (3.00%)
Total	57 (7.78%)	577 (78.72%)	43 (5.87%)	27 (3.69%)	29 (3.96%)	733 (100%)

### Engaging in Pup Play

Of the 733 participants, 527 participants (71.90%) identified as a pup; 71 (9.69%) identified as a handler; and 135 participants (18.42%) identified as both a pup and a handler (“switches”). Participants had identified with these roles for a mean duration of 4.29 years (*SD* = 4.90). A chi-square test of independence was performed to explore the relationship between chosen role (pup or handler) and the age group of participants. Switches were excluded. The relation between these variables was significant, *χ*^2^(5, *N* = 598) = 60.17, *p* < 0.001, with a medium effect size, *V* = 0.317. There is a positive association between being younger and identifying as a pup.

Most participants (*n* = 666, 90.9%) owned gear, with the most common pieces of gear being hoods/masks (*n* = 600, 81.86%), collars (*n* = 575, 78.44%) and harness/restraints (*n* = 463, 63.17%). Other gear included tails (*n* = 424, 57.84%), non-insertable objects mimicking dog toys (*n* = 416, 56.75%) and protective equipment (*n* = 344, 46.93%). Participants were asked how important gear was to them in expressing their pup identity on a 5-point Likert scale. A mean rating of 4 was given (*SD* = 1.14), with gear rated as “important” or “extremely important” to the majority of participants (*n* = 393, 53.61%), while just over one fifth (*n* = 167, 22.78%) stated gear was “not important.” This finding supports the notion that personal investment in pup play is important (Langdridge & Lawson, 2019; Newmahr, 2010), with participants potentially investing financial resources into pup play. It is also supported by the increasing availability of pup play gear sold in kink and fetish stores (Wignall, in press), including customizable pup hoods, which individuals can personalize to match their pup identity and make them more recognizable and unique.

Over half of participants (*n* = 372, 50.75%) stated they engaged in other fetishes during pup play. The most common fetishes were bondage (*n* = 193, 51.88%), watersports/urine play (*n* = 165, 44.35%) and impact play (normally where an individual is struck by another using either hands or objects) (*n* = 75, 20.16%). Other fetishes included wearing gear, chastity and fisting.

**Fig. 1:**
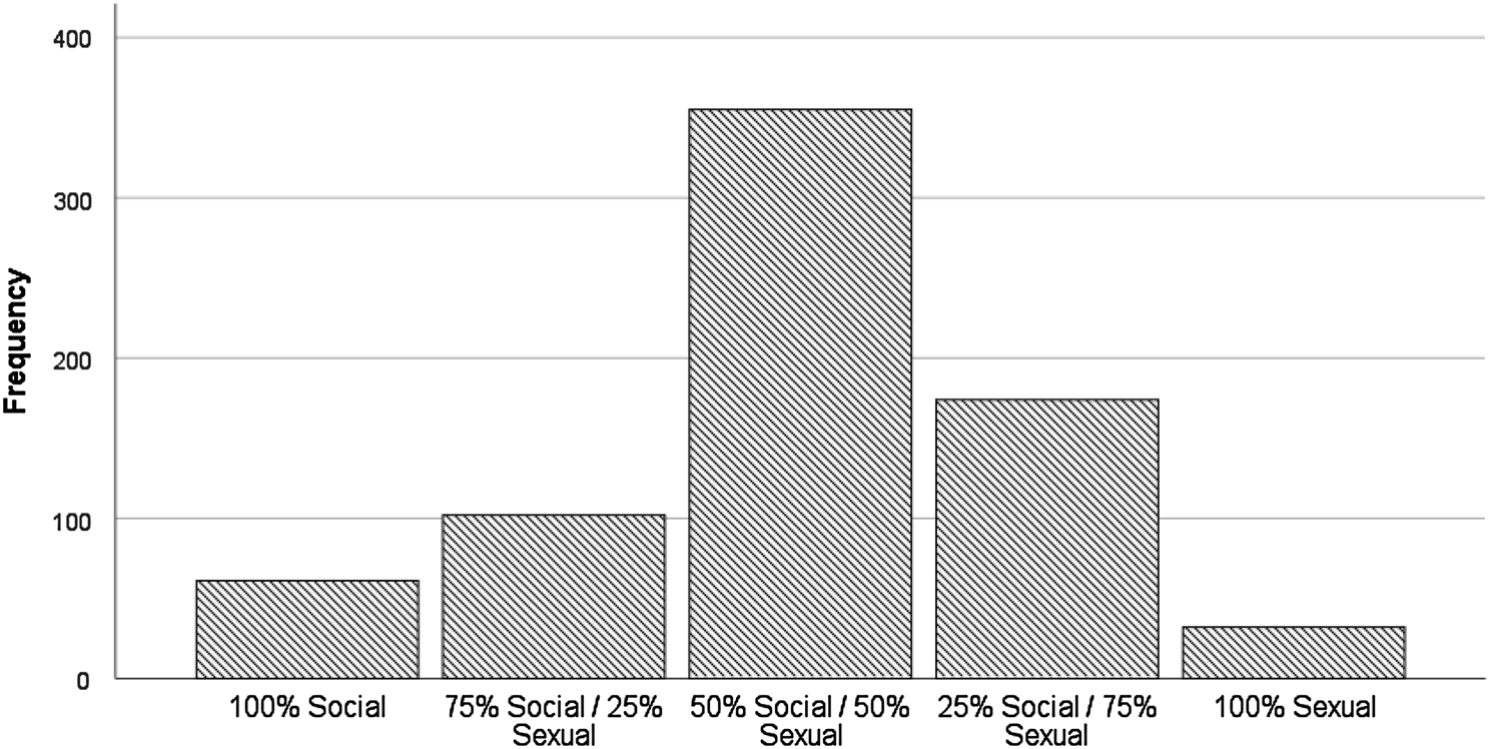
Participants’ framing of pup play as a social and/or sexual activity

Participants (*n* = 724) were asked the extent to which pup play was sexual and/or social, on a five-point scale from 100% Social to 100% Sexual. The mean response was 3.02 (*SD* = 0.95), with the majority (*n* = 355, 49.03%) rating their style of pup play as equally social and sexual (3 on the scale). The second most popular response was 75% sexual/25% social (n = 174, 24.03%) followed by 75% social / 25% sexual (*n* = 102, 14.09%). The ends of the scale, 100% social (n = 61, 8.43%) and 100% sexual (*n* = 32, 4.42%) contained a minority of participants (see Fig. 1).

Participants were asked about the role of community within their style of pup play. A majority of participants (*n* = 442, 60.30%) belonged to a related social group, often called Pups and Handler groups. The majority of participants had attended pup play oriented events. Two thirds had attended pup play themed events at public venues (*n* = 494, 67.4%), with 461 participants (62.89%) having attended a pup play “mosh” (group events where individuals engage in the social aspects of pup play only); one third of participants (*n* = 245, 33.42%) still attended moshes regularly. Just over one third of participants attended educational events and workshops, teaching how to do pup play (*n* = 271, 36.71%). This supports previous research which argued of the importance of community membership for those who engage in pup play (Langdridge & Lawson, 2019).

Almost one third of participants belonged to a pack/chosen family (*n* = 218, 29.74%). For those belonging to a pack, on a 5-point Likert scale ranging from extremely unimportant (1) to extremely important (5), participants gave a mean response of 4.1 (SD = 1.01), with three fourths describing their pack/chosen family as “very important” or “extremely important” (*n* = 166, 76.15%). For those who were not in a pack/chosen family (*n* = 515, 70.36%), almost half stated they want to join one in the future (*n* = 221, 42.91%) or might want to in the future (*n* = 232, 45.05%). Only 50 participants (6.82%) said they did not want to join a pack now or in the future.

Of the participants who identified as a pup [including switches] (*n* = 662, 90.31%), 574 participants (86.71%) had a chosen name for their pup personae, with this being chosen either by the respondent (*n* = 404, 61.03%), somebody close to them, such as handler or partner (*n* = 148, 22.36%), or a group of people (*n* = 21, 3.17%). Of those who identified as only a handler (*n* = 71), 38 (53.52%) also had a chosen name which related to their pup play identity.

Over half of participants who identified as pups identified with a dog breed (*n* = 363, 54.83%), with the most popular breed being Husky (*n* = 32, 8.82%), Wolf (*n* = 31, 8.54%) and German Shepherd (*n* = 27, 7.44%). 50 participants (13.77%) stated they were a mixture of dog breeds. Through creating a chosen name and identifying a particular dog breed, participants are arguably investing more in their pup identity, and it is notable that the most favored breeds are for large dogs—perhaps speaking to the intersection of masculinity with kink (Childs, 2016).

### Mental Health

Participants were asked if they had any mental health diagnoses: almost half of the sample did (*n* = 359, 49.00%), with 342 participants (46.66%) reporting they did not, 30 participants (4.09%) electing not to say and 2 participants (0.27%) not responding. Participants believed pup play improved their general mental health (*n* = 616, 84.04%); 71 participants (9.69%) stated that it does not.

A binary logistic regression was conducted to explore factors which predicted a mental health diagnosis. Analyses indicated that having a more social style of pup play and self-reporting of pup play improving mental health were significant predictors of having a mental health diagnosis [*χ*^2^ = 37.98, df = 4 and *p* < 0.001]. Belonging to a pack and personally identifying with the label pup/handler were not significant. All four predictors explained 7.6% [Nagelkerke R] of the variability of pup play improving mental health. Having a more social style of pup play and pup play improving mental health were significant at the 5% level [social style Wald = 15.83, *p* < 0.001; pup play improving mental health Wald = 12.00, *p* < 0.001]. The odds ratio (OR) for social style was 0.70 (95% CI 0.58–0.83), and for pup play improving mental health was 2.71 (95% CI: 1.54–4.76). The model correctly predicted 60.1% of cases.

A further binary logistic regression was conducted to explore factors which predicted perceived mental health benefits of pup play. Analyses indicated that having a mental health diagnosis, having a more social style of pup play, and personally identifying with the label pup/handler were significant predictors of self-reporting of pup play improving mental health [*χ*^2^ = 37.98, df = 4 and *p* < 0.001]. Belonging to a pack was not significant. All four predictors explain 11.6% [Nagelkerke R] of the variability of pup play improving mental health. Having a mental health diagnosis, having a more social style of pup play, and identifying with the term pup/handler were significant at the 5% level [mental health diagnosis Wald = 13.01, *p* < 0.001; social style Wald = 10.35, *p* = 0.001; identifying with the term Wald = 6.62, *p* = 0.10]. The odds ratio (OR) for mental health diagnosis was 4.77 (95% CI: 1.61–5.04), social style was 0.60 (95% CI 0.45–0.82), and for identifying with the term was 2.72 (95% CI: 1.27–5.83). The model correctly predicted 89.9% of cases.

## Discussion

This study has presented findings from a community survey of 733 respondents who engage in pup play, primarily from the US and with 30% of respondents from other countries. Supporting the conceptualization of pup play developed in the qualitative literature (Langdridge & Lawson, 2019; Wignall & McCormack, 2017), this study yields important information on characteristics of pup play due to its considerably larger sample size, use of quantitative data and diverse demographics primarily located in the US.

Regarding demographics, while the study supports the notion that pup play is predominantly practiced by gay and bisexual men, it also shows that it is practiced beyond these groups. Women, heterosexual men, and queer, trans and nonbinary individuals also participate in pup play. Similarly, while over half the sample are aged 18–30, there is still a wide age range of participants, including 41.88% over 30 years and 16.92% of participants aged over 40 years. The study also confirms the notion that pup play is practiced more by younger adults (Wignall & McCormack, 2017), with a significant association between belonging to an older age group and identifying as a handler. This may be the result of a combination of the physicality of pup play, such as crawling on knees and being active, being more accessible to younger adults, as well as norms related to age where subs being younger than doms is often privileged (Wignall, in press) and younger adults being in a period of life where sexual experimentation is more expected (Twenge, 2014). Thus, while the focus of research on pup play on young gay and bisexual male communities is not misplaced, future work needs to engage with the diversity of pup play practitioners and address how pup play occurs in different cultures and demographics.

The study also provides important information on the debate about the social and sexual nature of pup play and kink more generally (Wignall & McCormack, 2017). The great majority of respondents saw pup play as both social and sexual with less than ten percent saying it was exclusively social or sexual. This is significant as it supports the idea that pup play, and kink more generally, is a sociosexual activity. The findings on community features of pup play showed a similar trend—with a majority part of community PAH groups and two thirds having attended a pup play themed event. While these findings may be influenced by the sampling procedure, it supports both the arguments that pup play is fundamentally sexual and social as well as the critique that not all kink or pup play occurs in community settings and that research needs to reach out to people who are not part of such networks and practice kink primarily via hook up apps (Wignall, in press).

The study also develops our understanding of identity work related to pup play. Social identity theory has been used to understand individuals’ involvement in kinks, including pup play (Jaspal, 2019; Wignall, in press), and the widespread adoption of a name for pup personas alongside a majority associating with a dog breed supports this understanding of pup play being used to foster positive distinctiveness of social identity. Alongside this collective social identity, participants maintained uniqueness in their pup identity through symbols to represent their identity, including personalized pup hoods and gear. Such personal investment into these identities could pose risks for participants, potentially having to distance themselves from their pup identity due to social stigma related to pup play. Indeed, qualitative research has demonstrated how pups often conceal aspects of their pup identity or display discreet markers (Wignall, 2017).

It is notable that a majority of respondents reported a mental health diagnosis and that a large majority (84%) believed pup play improved their mental health. Our regression analyses found that having a mental health diagnosis was predicted by a more social style of pup play and the perception that it improved mental health. Perceived improvement in mental health was predicted by reporting a mental health diagnosis, having a more social style of pup play and identifying with the term. The self-report style of the survey and lack of information about the types of diagnoses make interpreting this finding difficult. Furthermore, the findings might also be an artifact of recruiting from community groups which may have people with elevated rates of mental health diagnosis (McCormack, 2014). Given the history of stigma and pathology with kink practices (Khan, 2014), care must be taken not to pathologize pup play but further investigate why these associations might occur. It might be, for example, that pup play is used as a form of self-directed therapy (Langdridge & Lawson, 2019), particularly given that many younger participants in particular are unlikely to have easy access to a therapist even though they may have suffered discrimination or stigma as a result of their sexual orientation and sexual interests more broadly (Meyer et al., 2021).

### Limitations and Conclusion

This study is not without its limitations. First, the sample is non-random and community based. This was necessary to gain sufficient responses in the context of the first large-scale quantitative survey of pup play. However, it limits generalizations that can be made from data with inherent biases and is likely to speak more to people engaged in pup play communities than those who engage in pup play outside of these networks. Future research should aim to recruit participants from various different kink networks to find people who are embedded within communities, as well as practicing kink more “casually” (see Prior & Williams, 2015; Wignall, in press).

There are also limitations in survey design. Demographic information, such as ethnicity, income and social class, were not collected as a result of the community-based design of the survey. Furthermore, the survey predominantly used questions with binary responses and free text boxes which limits the analyses. Future research should also include more sophisticated and validated scales of identity, mental health and community engagement to help build on the preliminary findings in this article. In the binary multiple regression predicting a mental health diagnosis and perceived metal health benefits of pup play, respectively, the amount of variance explained by the independent variables was relatively low. This suggests that additional factors may be accounting for the unexplained variance. It is therefore necessary to conduct further research introducing other possible independent variables.

In summary, this study uses a large data set with predominantly North American participants to provide support for the conceptualization of pup play as a kink activity documented in qualitative research. Our findings indicate that pup play is a sociosexual activity engaged in primarily by young gay men, but inclusive of diverse ages, genders and sexualities which mean it should not be seen as solely a pursuit of young gay men. Individuals forge unique identities within pup play, while also engaging with larger pup and other kink communities. Pup play is perceived to provide some mental health benefits for participants, but further research is needed to examine whether benefit exists beyond self-perception.

## Declarations

### Availability of Data and Material

For access to data, please email corresponding author.
